# 11-Butyl-3-meth­oxy-11*H*-benzo[*a*]carbazole

**DOI:** 10.1107/S1600536810021963

**Published:** 2010-06-16

**Authors:** Yavuz Ergün, Cevher Gündoğdu, Barış Tercan, Emel Ermiş, Tuncer Hökelek

**Affiliations:** a Department of Chemistry, Faculty of Arts and Sciences, Dokuz Eylül University, Tınaztepe, 35160 Buca, Izmir, Turkey; b Department of Physics, Karabük University, 78050 Karabük, Turkey; c Department of Chemistry, Faculty of Science, Anadolu University, 26470 Yenibağlar, Eskişehir, Turkey; d Department of Physics, Hacettepe University, 06800 Beytepe, Ankara, Turkey

## Abstract

The title compound, C_21_H_21_NO, consists of a carbazole skeleton with a meth­oxy­benzene ring fused to the carbazole, and a butyl group attached to the carbazole N atom. The carbazole skeleton is nearly planar [maximum deviation = 0.078 (2) Å], and it is oriented at a dihedral angle of 4.22 (4)° with respect to the adjacent meth­oxy­benzene ring.

## Related literature

For the biological activity of carbazole derivatives, see: Knölker & Reddy (2002 ▶). For the use of carbazole derivatives in the syntheses of indole alkaloids, see: Routier *et al.* (2001 ▶). For the use of benzo[*a*]carbazoles in cancer treatment, see: Carini *et al.* (2001 ▶). For the anti­tumor activity of a series of simple benzo[*a*]carbazoles against mammary tumors of rats, leukemia, renal tumors, colon cancer and malignant melanoma tumor cell lines, see: von Angerer & Prekajac (1986 ▶); Pindur & Lemster (1997 ▶). For the extensive application of benzo[*a*]carbazole derivatives as photographic materials, see: Oliveira *et al.* (2005 ▶. For tetra­hydro­carbazole systems present in the frameworks of a number of indole-type alkaloids of biological inter­est, see: Phillipson & Zenk (1980 ▶); Saxton (1983 ▶); Abraham (1975 ▶). For related structures, see: Hökelek *et al.* (1994 ▶, 1998 ▶, 1999 ▶, 2004 ▶, 2006 ▶); Patır *et al.* (1997 ▶); Hökelek & Patır (1999 ▶, 2002 ▶); Çaylak *et al.* (2007 ▶). For bond-length data, see: Allen *et al.* (1987 ▶).
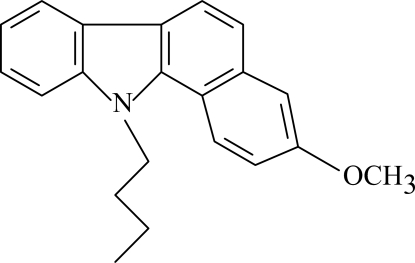


## Experimental

### 

#### Crystal data

C_21_H_21_NO
*M* *_r_* = 303.39
Monoclinic, 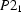

*a* = 10.7263 (6) Å
*b* = 5.5562 (3) Å
*c* = 13.8967 (7) Å
β = 97.841 (2)°
*V* = 820.46 (8) Å^3^
*Z* = 2
Mo *K*α radiation
μ = 0.08 mm^−1^
*T* = 100 K
0.48 × 0.39 × 0.35 mm

#### Data collection

Bruker Kappa APEXII CCD area-detector diffractometer
Absorption correction: multi-scan (*SADABS*; Bruker, 2005 ▶) *T*_min_ = 0.965, *T*_max_ = 0.974
7947 measured reflections
2248 independent reflections
2001 reflections with *I* > 2σ(*I*)
*R*_int_ = 0.027

#### Refinement

*R*[*F* ^2^ > 2σ(*F* ^2^)] = 0.035
*wR*(*F* ^2^) = 0.091
*S* = 1.04
2248 reflections
270 parameters
H atoms treated by a mixture of independent and constrained refinement
Δρ_max_ = 0.25 e Å^−3^
Δρ_min_ = −0.16 e Å^−3^

### 

Data collection: *APEX2* (Bruker, 2007 ▶); cell refinement: *SAINT* (Bruker, 2007 ▶); data reduction: *SAINT*; program(s) used to solve structure: *SHELXS97* (Sheldrick, 2008 ▶); program(s) used to refine structure: *SHELXL97* (Sheldrick, 2008 ▶); molecular graphics: *ORTEP-3 for Windows* (Farrugia, 1997 ▶); software used to prepare material for publication: *WinGX* (Farrugia, 1999 ▶).

## Supplementary Material

Crystal structure: contains datablocks I, global. DOI: 10.1107/S1600536810021963/xu2773sup1.cif

Structure factors: contains datablocks I. DOI: 10.1107/S1600536810021963/xu2773Isup2.hkl

Additional supplementary materials:  crystallographic information; 3D view; checkCIF report

## Figures and Tables

**Fig. 1.**
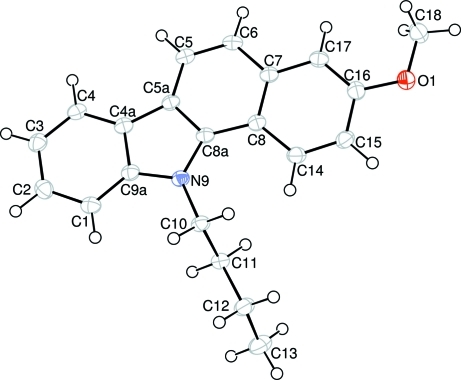
The molecular structure of the title molecule with the atom-numbering scheme. The displacement ellipsoids are drawn at the 50% probability level.

**Fig. 2.**
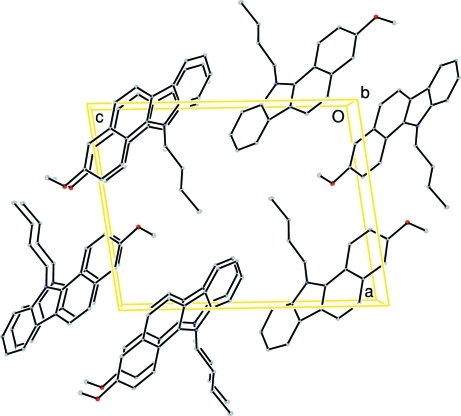
A partial packing diagram.

## supplementary crystallographic information

### Comment

Carbazole derivatives display a range of biological activities, making them attractive compounds to synthetic and medicinal chemists (Knölker & Reddy, 2002). They also have an important role in the syntheses of indole alkaloids, in which their synthetic derivatives, possessing useful pharmacological properties, are currently under investigation (Routier *et al.*, 2001). Benzo -annulated carbazole ring systems are found only rarely in natural products. The benzo[*a*]carbazoles, containing an aromatic ring fused to the a-face of the carbazole nucleus, are potential candidates for cancer treatment as a result of DNA intercelative binding properties (Carini *et al.*, 2001). A series of simple benzo[*a*]carbazoles have been shown to bind to estrogen receptors and inhibit the growth of mammary tumors of rats (Angerer & Prekajac, 1986). Some benzo[*a*]carbazoles exhibit a pronounced antitumor activity against leukemia, renal tumor, colon cancer, and malignant melanoma tumor cell lines (Pindur & Lemster, 1997). Benzo[*a*]carbazole derivatives have also found extensive application as photographic materials (Oliveira *et al.*, 2005).

Tetrahydrocarbazole systems are present in the framework of a number of indole-type alkaloids of biological interest (Phillipson & Zenk, 1980; Saxton, 1983; Abraham, 1975). The structures of tricyclic, tetracyclic and pentacyclic ring systems with dithiolane and other substituents of the tetrahydrocarbazole core, have been the subject of much interest in our laboratory. These include 1,2,3,4-tetrahydrocarbazole-1-spiro-2'-[1,3]dithiolane, (II) (Hökelek *et al.*, 1994), *N*-(2-methoxyethyl)-*N*-{2,3,4,9-tetrahydrospiro[1*H*-carbazole-1, 2-(1,3)dithiolane]-4-yl}benzene-sulfonamide, (III) (Patır *et al.*, 1997), spiro[carbazole-1(2*H*),2'-[1,3]-dithiolan]-4(3*H*)-one, (IV) (Hökelek *et al.*, 1998), 9-acetonyl-3-ethylidene-1,2,3,4-tetrahydrospiro[carbazole-1,2'-[1,3] dithiolan]-4-one, (V) (Hökelek *et al.*, 1999), *N*-(2,2-dimethoxyethyl)-N -{9-methoxymethyl-1,2,3,4-tetrahydrospiro[carbazole-1,2'-[1,3]dithiolan] -4-yl}benzamide, (VI) (Hökelek & Patır, 1999), 3a,4,10,10*b*-tetrahydro-2H -furo[2,3-*a*]carbazol-5(3*H*)-one, (VII) (Çaylak *et al.*, 2007); also the pentacyclic compounds 6-ethyl-4-(2-methoxyethyl)-2,6-methano-5-oxo-hexahydro- pyrrolo(2,3 - d)carbazole-1-spiro-2'-(1,3)dithiolane, (VIII) (Hökelek & Patır, 2002), *N*-(2-benzyloxyethyl)-4,7-dimethyl-6-(1,3-dithiolan-2-yl)-1,2, 3,4,5,6-hexahydro-1,5-methano-2-azocino[4,3-*b*]indol-2-one, (IX) (Hökelek *et al.*, 2004) and 4-ethyl-6,6-ethylenedithio-2-(2-methoxyethyl)-7-methoxy- methylene-2,3,4,5,6,7-hexahydro-1,5-methano-1*H*-azocino[4,3-*b*]indol-3-one, (*X*) (Hökelek *et al.*, 2006). The title compound, (I), may be considered as a synthetic precursor of tetracyclic indole alkaloids of biological interests. The present study was undertaken to ascertain its crystal structure.

The title compound consists of a carbazole skeleton with a methoxy benzoato group fused to the a-face of the carbazole nucleus, and a butyl group attached to atom N9 (Fig. 1), where the bond lengths (Allen *et al.*, 1987) and angles are within normal ranges, and generally agree with those in compounds (II)-(*X*). In all structures atom N9 is substituted.

An examination of the deviations from the least-squares planes through individual rings shows that rings A (C1—C4/C4a/C9a), B (C4a/C5a/C8a/N9/C9a), C (C5a/C5—C8/C8a) and D (C7/C8/C14—C17) are planar. The carbazole skeleton, containing the rings A, B and C are also nearly planar [with a maximum deviation of 0.078 (2) Å for atom C2] with dihedral angles of A/B = 2.37 (6), A/C = 5.01 (5) and B/C = 2.81 (5) ° Ring D is oriented with respect to the planar carbazole skeleton at a dihedral angle of 4.22 (4) °. So, it is nearly coplanar with the carbazole skeleton. Atoms O1 and C18 displaced by 0.010 (1) and -0.045 (2) Å from the plane of ring D, respectively, while atom C10 is 0.092 (2) Å away from the plane of the carbazole skeleton.

In the crystal structure, molecules are alongated along the *c* axis and stacked along the *b* axis (Fig. 2).

### Experimental

For the preparation of the title compound, (I), a solution of 3-methoxy-11H -benzo[*a*]carbazole (1.00 g, 4.0 mmol) in dichloromethane (20 ml) was cooled to 273 K, and then sodium hydroxide (2 ml, 50%), tetrabutylammonium hydrogen sulfate (0.10 g) and butyl bromide (0.62 g, 4.5 mmol) were added. The mixture was stirred for 1 h at 273 K, and then 2 h at 298 K. It was washed with hydrochloric acid (50 ml, 10%) and the organic layer was dried with anhydrous magnesium sulfate. The solvent was evaporated under reduced pressure and the residue was crystallized from methanol (yield; 1.15 g, 93%, m.p. 369 K).

### Refinement

H13A, H13B, H13C, H18A, H18B and H18C atoms (for methyl groups) were positioned geometrically with C—H = 0.98 Å and constrained to ride on their parent atoms, *U*_iso_(H) = 1.5*U*_eq_(C). The remaining H atoms were located in difference Fourier maps and refined isotropically. Friedel pairs were merged because of the weak anomalous scatterer of the compound, and the absolute structure was not determined.

### Crystal data

**Table:**

C_21_H_21_NO	*F*(000) = 324
*M**_r_* = 303.39	*D*_x_ = 1.228 Mg m^−^^3^
Monoclinic, *P*2_1_	Mo *K*α radiation, λ = 0.71073 Å
Hall symbol: P 2yb	Cell parameters from 3840 reflections
*a* = 10.7263 (6) Å	θ = 2.3–28.4°
*b* = 5.5562 (3) Å	µ = 0.08 mm^−^^1^
*c* = 13.8967 (7) Å	*T* = 100 K
β = 97.841 (2)°	Block, colorless
*V* = 820.46 (8) Å^3^	0.48 × 0.39 × 0.35 mm
*Z* = 2	

### Data collection

**Table:**

Bruker Kappa APEXII CCD area-detector diffractometer	2248 independent reflections
Radiation source: fine-focus sealed tube	2001 reflections with *I* > 2σ(*I*)
graphite	*R*_int_ = 0.027
φ and ω scans	θ_max_ = 28.4°, θ_min_ = 1.5°
Absorption correction: multi-scan (*SADABS*; Bruker, 2005)	*h* = −13→14
*T*_min_ = 0.965, *T*_max_ = 0.974	*k* = −7→6
7947 measured reflections	*l* = −17→18

### Refinement

**Table:**

Refinement on *F*^2^	Secondary atom site location: difference Fourier map
Least-squares matrix: full	Hydrogen site location: inferred from neighbouring sites
*R*[*F*^2^ > 2σ(*F*^2^)] = 0.035	H atoms treated by a mixture of independent and constrained refinement
*wR*(*F*^2^) = 0.091	*w* = 1/[σ^2^(*F*_o_^2^) + (0.0528*P*)^2^ + 0.1088*P*] where *P* = (*F*_o_^2^ + 2*F*_c_^2^)/3
*S* = 1.03	(Δ/σ)_max_ < 0.001
2248 reflections	Δρ_max_ = 0.25 e Å^−^^3^
270 parameters	Δρ_min_ = −0.16 e Å^−^^3^
Primary atom site location: structure-invariant direct methods	

### Special details

**Table:**

Geometry. All e.s.d.'s (except the e.s.d. in the dihedral angle between two l.s. planes) are estimated using the full covariance matrix. The cell e.s.d.'s are taken into account individually in the estimation of e.s.d.'s in distances, angles and torsion angles; correlations between e.s.d.'s in cell parameters are only used when they are defined by crystal symmetry. An approximate (isotropic) treatment of cell e.s.d.'s is used for estimating e.s.d.'s involving l.s. planes.
Refinement. Refinement of *F*^2^ against ALL reflections. The weighted *R*-factor *wR* and goodness of fit *S* are based on *F*^2^, conventional *R*-factors *R* are based on *F*, with *F* set to zero for negative *F*^2^. The threshold expression of *F*^2^ > σ(*F*^2^) is used only for calculating *R*-factors(gt) *etc*. and is not relevant to the choice of reflections for refinement. *R*-factors based on *F*^2^ are statistically about twice as large as those based on *F*, and *R*- factors based on ALL data will be even larger.

### Fractional atomic coordinates and isotropic or equivalent isotropic displacement parameters (Å^2^)

**Table:**

	*x*	*y*	*z*	*U*_iso_*/*U*_eq_	
O1	0.59965 (11)	0.9420 (3)	−0.11895 (9)	0.0299 (3)	
C1	1.02958 (17)	0.2221 (4)	0.41676 (13)	0.0246 (4)	
H1	0.9764 (19)	0.094 (5)	0.4258 (15)	0.030 (6)*	
C2	1.14448 (17)	0.2475 (4)	0.47538 (13)	0.0276 (4)	
H2	1.166 (2)	0.136 (5)	0.5266 (17)	0.042 (7)*	
C3	1.22609 (17)	0.4381 (4)	0.46185 (13)	0.0271 (4)	
H3	1.3035 (19)	0.447 (4)	0.5040 (14)	0.022 (5)*	
C4	1.19492 (16)	0.6061 (4)	0.38921 (13)	0.0245 (4)	
H4	1.2520 (17)	0.742 (4)	0.3796 (13)	0.021 (5)*	
C4A	1.08080 (15)	0.5806 (4)	0.32759 (12)	0.0217 (4)	
C5	1.06152 (16)	0.9086 (4)	0.19246 (13)	0.0232 (4)	
H5	1.1411 (18)	0.992 (4)	0.2181 (13)	0.020 (5)*	
C5A	1.02208 (15)	0.7091 (3)	0.24338 (12)	0.0210 (4)	
C6	0.98899 (16)	0.9848 (4)	0.10954 (13)	0.0238 (4)	
H6	1.0136 (18)	1.120 (5)	0.0755 (14)	0.025 (5)*	
C7	0.87121 (15)	0.8724 (4)	0.07581 (12)	0.0215 (4)	
C8	0.82600 (15)	0.6754 (4)	0.12711 (12)	0.0205 (4)	
C8A	0.90768 (15)	0.5921 (4)	0.21092 (12)	0.0197 (3)	
C9A	0.99973 (15)	0.3887 (4)	0.34259 (12)	0.0214 (4)	
N9	0.89451 (13)	0.3987 (3)	0.27259 (10)	0.0209 (3)	
C10	0.79576 (15)	0.2165 (4)	0.26464 (13)	0.0221 (4)	
H101	0.8331 (18)	0.076 (5)	0.2927 (14)	0.021 (5)*	
H102	0.7675 (17)	0.185 (4)	0.1949 (14)	0.021 (5)*	
C11	0.68345 (16)	0.2861 (4)	0.31519 (13)	0.0228 (4)	
H111	0.7121 (18)	0.304 (4)	0.3868 (14)	0.023 (5)*	
H112	0.6483 (19)	0.442 (5)	0.2886 (15)	0.027 (6)*	
C12	0.58043 (17)	0.0964 (4)	0.29886 (15)	0.0290 (4)	
H121	0.6196 (19)	−0.064 (5)	0.3171 (14)	0.025 (5)*	
H122	0.5517 (19)	0.077 (5)	0.2304 (17)	0.036 (6)*	
C13	0.47038 (18)	0.1518 (5)	0.35479 (16)	0.0397 (5)	
H13A	0.4050	0.0290	0.3400	0.060*	
H13B	0.4355	0.3104	0.3355	0.060*	
H13C	0.5002	0.1516	0.4247	0.060*	
C14	0.70388 (16)	0.5850 (4)	0.09343 (13)	0.0248 (4)	
H14	0.668 (2)	0.458 (5)	0.1282 (15)	0.032 (6)*	
C15	0.63327 (16)	0.6779 (4)	0.01272 (13)	0.0264 (4)	
H15	0.549 (2)	0.613 (5)	−0.0092 (17)	0.044 (7)*	
C16	0.68014 (16)	0.8672 (4)	−0.03944 (12)	0.0242 (4)	
C17	0.79658 (16)	0.9641 (4)	−0.00876 (12)	0.0235 (4)	
H17	0.8282 (16)	1.097 (4)	−0.0415 (13)	0.015 (5)*	
C18	0.63988 (18)	1.1414 (4)	−0.17108 (14)	0.0325 (5)	
H18A	0.5741	1.1833	−0.2245	0.049*	
H18B	0.7172	1.0988	−0.1974	0.049*	
H18C	0.6561	1.2794	−0.1272	0.049*	

### Atomic displacement parameters (Å^2^)

**Table:**

	*U*^11^	*U*^22^	*U*^33^	*U*^12^	*U*^13^	*U*^23^
O1	0.0241 (6)	0.0399 (9)	0.0257 (6)	0.0017 (6)	0.0027 (5)	0.0051 (7)
C1	0.0267 (9)	0.0213 (10)	0.0275 (8)	0.0008 (8)	0.0096 (7)	−0.0004 (8)
C2	0.0292 (9)	0.0284 (11)	0.0259 (9)	0.0058 (8)	0.0057 (7)	0.0023 (8)
C3	0.0228 (8)	0.0342 (12)	0.0250 (8)	0.0021 (8)	0.0062 (7)	−0.0029 (9)
C4	0.0212 (8)	0.0280 (11)	0.0253 (8)	−0.0024 (8)	0.0071 (6)	−0.0025 (8)
C4A	0.0222 (8)	0.0219 (9)	0.0223 (8)	0.0003 (7)	0.0077 (6)	−0.0039 (8)
C5	0.0215 (8)	0.0229 (9)	0.0263 (8)	−0.0032 (7)	0.0075 (7)	−0.0025 (8)
C5A	0.0197 (7)	0.0218 (9)	0.0229 (8)	0.0002 (7)	0.0072 (6)	−0.0024 (7)
C6	0.0262 (8)	0.0209 (9)	0.0261 (9)	−0.0032 (8)	0.0100 (7)	0.0006 (8)
C7	0.0215 (8)	0.0226 (10)	0.0220 (8)	0.0009 (7)	0.0088 (7)	−0.0025 (7)
C8	0.0207 (7)	0.0203 (9)	0.0215 (8)	0.0008 (7)	0.0065 (6)	−0.0024 (7)
C8A	0.0203 (7)	0.0178 (9)	0.0225 (8)	0.0001 (7)	0.0081 (6)	−0.0021 (7)
C9A	0.0199 (7)	0.0217 (9)	0.0239 (8)	0.0016 (7)	0.0076 (6)	−0.0015 (7)
N9	0.0204 (6)	0.0186 (8)	0.0240 (7)	−0.0017 (6)	0.0045 (5)	0.0000 (6)
C10	0.0210 (8)	0.0189 (9)	0.0269 (9)	−0.0034 (7)	0.0051 (7)	−0.0001 (8)
C11	0.0212 (8)	0.0226 (10)	0.0252 (9)	−0.0026 (7)	0.0054 (6)	0.0003 (7)
C12	0.0230 (8)	0.0338 (12)	0.0307 (10)	−0.0073 (8)	0.0051 (7)	−0.0015 (9)
C13	0.0246 (9)	0.0471 (15)	0.0491 (12)	−0.0051 (10)	0.0112 (8)	0.0041 (11)
C14	0.0247 (8)	0.0265 (10)	0.0240 (8)	−0.0033 (8)	0.0062 (6)	0.0006 (8)
C15	0.0201 (8)	0.0323 (11)	0.0272 (9)	−0.0018 (8)	0.0049 (7)	−0.0012 (8)
C16	0.0235 (8)	0.0286 (10)	0.0211 (8)	0.0045 (7)	0.0051 (7)	−0.0009 (8)
C17	0.0246 (8)	0.0240 (10)	0.0234 (8)	0.0005 (7)	0.0087 (6)	−0.0008 (8)
C18	0.0324 (9)	0.0366 (12)	0.0283 (9)	0.0040 (9)	0.0035 (8)	0.0064 (9)

### Geometric parameters (Å, °)

**Table:**

O1—C16	1.371 (2)	C10—C11	1.525 (2)
O1—C18	1.422 (3)	C10—H101	0.94 (2)
C1—C2	1.389 (3)	C10—H102	0.991 (19)
C1—H1	0.93 (2)	C11—C12	1.521 (3)
C2—C3	1.403 (3)	C11—H111	1.006 (19)
C2—H2	0.95 (3)	C11—H112	0.99 (3)
C3—C4	1.382 (3)	C12—C13	1.530 (3)
C3—H3	0.95 (2)	C12—H121	1.00 (2)
C4—H4	0.99 (2)	C12—H122	0.97 (2)
C4A—C4	1.402 (2)	C13—H13A	0.9800
C4A—C9A	1.410 (3)	C13—H13B	0.9800
C5—C5A	1.411 (3)	C13—H13C	0.9800
C5—H5	1.00 (2)	C14—C8	1.421 (2)
C5A—C4A	1.440 (2)	C14—H14	0.97 (2)
C6—C5	1.367 (3)	C15—C14	1.366 (3)
C6—H6	0.95 (2)	C15—H15	0.98 (2)
C7—C6	1.430 (2)	C16—C15	1.408 (3)
C8—C7	1.427 (2)	C16—C17	1.373 (3)
C8—C8A	1.435 (2)	C17—C7	1.423 (3)
C8A—C5A	1.408 (2)	C17—H17	0.96 (2)
C9A—C1	1.390 (3)	C18—H18A	0.9800
N9—C8A	1.393 (2)	C18—H18B	0.9800
N9—C9A	1.387 (2)	C18—H18C	0.9800
N9—C10	1.458 (2)		
			
C16—O1—C18	116.45 (15)	N9—C10—H101	106.4 (12)
C2—C1—C9A	117.62 (18)	N9—C10—H102	108.7 (12)
C2—C1—H1	120.6 (13)	C11—C10—H101	109.8 (12)
C9A—C1—H1	121.7 (13)	C11—C10—H102	109.9 (11)
C1—C2—C3	121.20 (18)	H101—C10—H102	108.3 (19)
C1—C2—H2	119.0 (15)	C10—C11—H111	109.1 (11)
C3—C2—H2	119.8 (15)	C10—C11—H112	109.4 (12)
C2—C3—H3	117.8 (14)	C12—C11—C10	111.04 (16)
C4—C3—C2	120.97 (17)	C12—C11—H111	109.5 (13)
C4—C3—H3	121.2 (14)	C12—C11—H112	108.4 (13)
C3—C4—C4A	118.82 (18)	H112—C11—H111	109.4 (18)
C3—C4—H4	121.4 (11)	C11—C12—C13	112.32 (18)
C4A—C4—H4	119.8 (11)	C11—C12—H121	107.9 (12)
C4—C4A—C5A	134.01 (18)	C11—C12—H122	110.6 (15)
C4—C4A—C9A	119.40 (17)	C13—C12—H121	112.1 (12)
C9A—C4A—C5A	106.56 (14)	C13—C12—H122	111.0 (13)
C5A—C5—H5	119.3 (12)	H121—C12—H122	103 (2)
C6—C5—C5A	119.41 (17)	C12—C13—H13A	109.5
C6—C5—H5	121.3 (12)	C12—C13—H13B	109.5
C5—C5A—C4A	132.01 (16)	C12—C13—H13C	109.5
C8A—C5A—C4A	107.26 (16)	H13A—C13—H13B	109.5
C8A—C5A—C5	120.68 (16)	H13A—C13—H13C	109.5
C5—C6—C7	121.13 (17)	H13B—C13—H13C	109.5
C5—C6—H6	120.2 (12)	C8—C14—H14	120.6 (13)
C7—C6—H6	118.5 (12)	C15—C14—C8	121.32 (18)
C8—C7—C6	121.11 (16)	C15—C14—H14	118.1 (13)
C17—C7—C6	119.12 (17)	C14—C15—C16	120.52 (17)
C17—C7—C8	119.75 (16)	C14—C15—H15	120.0 (16)
C7—C8—C8A	116.20 (15)	C16—C15—H15	119.5 (15)
C14—C8—C7	117.85 (17)	O1—C16—C15	114.31 (16)
C14—C8—C8A	125.91 (17)	O1—C16—C17	125.26 (18)
C5A—C8A—C8	121.33 (16)	C17—C16—C15	120.42 (17)
N9—C8A—C5A	108.44 (15)	C7—C17—H17	118.4 (11)
N9—C8A—C8	130.23 (16)	C16—C17—C7	120.08 (18)
C1—C9A—C4A	121.96 (16)	C16—C17—H17	121.5 (11)
N9—C9A—C1	129.06 (17)	O1—C18—H18A	109.5
N9—C9A—C4A	108.98 (15)	O1—C18—H18B	109.5
C8A—N9—C10	128.47 (14)	O1—C18—H18C	109.5
C9A—N9—C8A	108.74 (14)	H18A—C18—H18B	109.5
C9A—N9—C10	122.57 (15)	H18A—C18—H18C	109.5
N9—C10—C11	113.60 (15)	H18B—C18—H18C	109.5
			
C18—O1—C16—C15	176.75 (16)	N9—C8A—C5A—C4A	0.40 (19)
C18—O1—C16—C17	−2.2 (3)	N9—C8A—C5A—C5	178.18 (16)
C9A—C1—C2—C3	1.6 (3)	C8—C8A—C5A—C4A	−179.64 (15)
C1—C2—C3—C4	−0.4 (3)	C8—C8A—C5A—C5	−1.9 (3)
C2—C3—C4—C4A	−1.2 (3)	N9—C9A—C1—C2	177.47 (17)
C5A—C4A—C4—C3	−176.09 (19)	C4A—C9A—C1—C2	−1.3 (3)
C9A—C4A—C4—C3	1.5 (2)	C9A—N9—C8A—C5A	−1.06 (18)
C4—C4A—C9A—N9	−179.24 (16)	C9A—N9—C8A—C8	178.97 (17)
C4—C4A—C9A—C1	−0.2 (2)	C10—N9—C8A—C5A	−175.70 (16)
C5A—C4A—C9A—N9	−1.06 (18)	C10—N9—C8A—C8	4.3 (3)
C5A—C4A—C9A—C1	177.96 (16)	C8A—N9—C9A—C1	−177.60 (17)
C6—C5—C5A—C4A	175.63 (18)	C8A—N9—C9A—C4A	1.33 (18)
C6—C5—C5A—C8A	−1.5 (3)	C10—N9—C9A—C1	−2.6 (3)
C5—C5A—C4A—C4	0.8 (3)	C10—N9—C9A—C4A	176.34 (15)
C5—C5A—C4A—C9A	−177.03 (18)	C9A—N9—C10—C11	96.69 (19)
C8A—C5A—C4A—C4	178.20 (19)	C8A—N9—C10—C11	−89.3 (2)
C8A—C5A—C4A—C9A	0.40 (19)	N9—C10—C11—C12	176.39 (15)
C7—C6—C5—C5A	2.7 (3)	C10—C11—C12—C13	175.83 (17)
C8—C7—C6—C5	−0.6 (3)	C15—C14—C8—C7	2.1 (3)
C17—C7—C6—C5	177.50 (17)	C15—C14—C8—C8A	179.73 (17)
C8A—C8—C7—C6	−2.6 (2)	C16—C15—C14—C8	−0.1 (3)
C8A—C8—C7—C17	179.29 (16)	O1—C16—C15—C14	179.77 (17)
C14—C8—C7—C6	175.24 (16)	C17—C16—C15—C14	−1.3 (3)
C14—C8—C7—C17	−2.8 (2)	O1—C16—C17—C7	179.31 (16)
C7—C8—C8A—N9	−176.22 (16)	C15—C16—C17—C7	0.5 (3)
C7—C8—C8A—C5A	3.8 (2)	C16—C17—C7—C6	−176.50 (16)
C14—C8—C8A—N9	6.1 (3)	C16—C17—C7—C8	1.6 (3)
C14—C8—C8A—C5A	−173.86 (17)		
